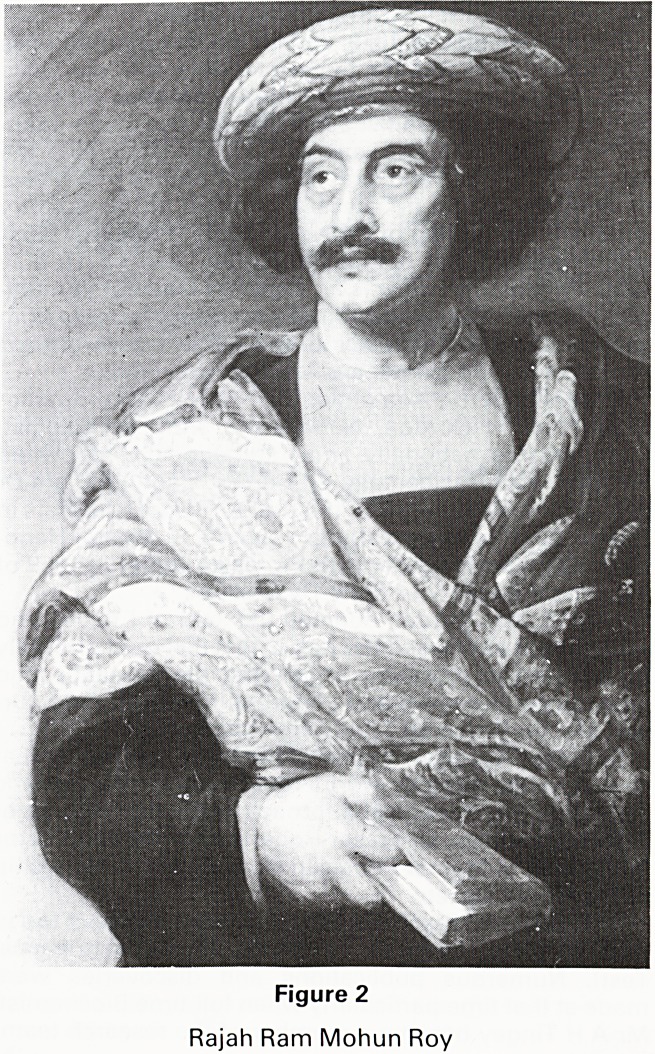# The History of Mental Handicap in Bristol and Bath (Part 2)

**Published:** 1986-08

**Authors:** J Jancar

**Affiliations:** Consultant Psychiatrist, Stoke Park Group of Hospitals


### History of Mental Handicap in Bristol and Bath Part II

**J Jancar M.B., B.Ch., B.A.O., F.R.C.Psych., D.P.M.**
Consultant Psychiatrist, Stoke Park Group of Hospitals

### LEIGH COURT HOSPITAL

Leigh Court also has a very interesting history. It was called Lege in the Domesday Book. The Manor of Leigh at one time belonged to the Monastery of St Augustine at Bristol. After the dissolution of the Monastery, it was passed to Paul Bush the first Bishop of Bristol, and afterwards by grant of the King to Sir George Norton.

On 16 September 1651 the Nortons gave shelter for four nights to King Charles I after his defeat at Worcester. In January 1884 the late King Edward VII, then Prince of Wales, was entertained by the late Sir Philip Miles at Leigh Court.

Another note of interest about Leigh Court relates to Dame Grace Gethin, wife of Sir Richard Gethin, who was the last surviving member of the Norton family from Leigh Court. As benefactress, she is remembered by the bestowal of the 'Gethin Shilling' on a number of widows at Westminster Abbey in a Lenten ceremony on Ash Wednesday. The Sermon and Commemoration of Dame Grace still continue but the bread charity has ceased.

In the south aisle of Westminster Abbey stands an elaborate memorial to Grace Gethin, erected by her parents. In 1700 a book under the title 'Reliquiae Gethinanae' was published and is preserved in the North Library of the British Museum.

Isaac D'Israeli, scholarly father of Queen Victoria's favourite statesman, had an irresistible urge to collect curiosities presented by literature. Early in the 19th century, alerted by the marble book at Westminster, he traced the real book and was quick to recognise Bacon's writings in it. He did not locate all that was Bacon's but suspected among what was left unacknowledged quotations from the works of Owen Feltham and Francis Osborne, D'Israeli considered further investigation unnecessary. There can be little doubt that the existence of the book was due simply to Grace Gethin's habit of copying passages that had impressed her in books that she read, so that she might better remember them. After her death, numerous loose sheets were found covered with her handwriting, and on these the presumption of authorship was founded. Therefore Grace Gethin was never party to such gross plagiarism. She was the victim of the excessive devotion and the limited literacy of her parents and friends (Jancar, 1974). Leigh Court was closed this year and sold. It is pleasing that it was bought by a voluntary organisation and again is providing accommodation for Mentally Handicapped people.

**Figure 1**

Stapleton Grove (Beech House)

### STAPLETON GROVE (Beech House)

Stapleton Grove (Fig 1) was built by Joseph Harford in about 1763. He was Sheriff of Bristol in 1779, Lord Mayor Elect in 1794 and a close friend of Edmund Burke. Henry Charles Harford sold Stapleton Grove to the Castle family in 1832. After the death of Mr and Mrs Castle in 1833, and on the invitation of Mary Carpenter, Rajah Ram Mohun Roy, (Fig 2) an Indian religious and social leader, a fighter for the abolition of Suttee and founder of Brahma Somaj,

**Figure 2**

Rajah Ram Mohun Roy stayed in Stapleton Grove until he died from meningitis in September 1833. He was buried in the Stapleton Grove grounds and ten years later interred at Arnos Vale Cemetery. After that the house was occupied by the Rector of Stapleton Church, Bishop Morell and was also at one time used as a boys' school.

### PURDOWN HOSPITAL—HEATH HOUSE

The first known deed relating to Heath House is dated 1425. Heath House belonged to the religious order in Bristol known as the Hospital of St Bartholomew. Just before the dissolution of the monasteries by Henry VIII, Heath House was sold to Robert Thorne, a merchant of Bristol. In 1546, the Walters family became tenants of the house, and their successors lived there for five generations. In 1767, through marriage, the Smythe family of Ashton Court, Bristol became owners of Heath House until 1911, when it was bought by the Reverend H N Burden.

### HANHAM HALL HOSPITAL

Hanham Hall was built in 1655 by Richard Jones. On his death in 1697, it became the property of Thomas Tyre. Hanham Hall changed ownership again in 1791 and in 1803 when it was purchased by the Whittuck family who stayed there until 1916. The finest feature of Hanham Hall is a very good example of an early 18th century shell-headed main entrace with flanking niches.

Mrs Burden died in 1919, and Mr Burden married Miss R Williams, who was the Superintendent of Stoke Park.

Mr Burden was not content merely to offer custodial care for Mentally Handicapped patients but made financial provision for and encouraged research into the causes, treatment and prevention of Mental Handicap. He established the first research centre in this country under the leadership of Professor Berry. Professor Berry was appointed just before Mr Burden died on 15 May 1930. Professor Berry was an excellent leader and multidisciplinarian and he appointed a number of people from various spheres of medicine and formed a very good relationship with Bristol and London Universities.

Professor Berry, Anatomist, Dr Norman, Neuropathologist and Dr Gordon, Neurologist and Psychologist, soon became nationally and internationally renowned for their work. Incidentally Dr Gordon's son, the late Dr Ian Gordon, Radiologist at the United Bristol Hospitals in Bristol, contributed greatly to research in Mental Handicap. He was co-founder with Professor Butler in 1969 of the Bristol Registry of Bone Dysplasias.

In 1933, Mrs Burden donated the sum of £10,000 and with the gift expressed her desire that it should primarily be devoted to problems underlying the causation and inheritance of normal and abnormal mentality. The Burden Mental Research Trust came into being.

Dr Fraser-Roberts was one of the leading minds at that time and he became world renowned for his genetic approach, psychological approach and other studies of Mental Handicap. Dr Fraser-Roberts was the first doctor in the field of Mental Handicap to receive the Fellowship of the Royal Society.

Dr R Griffiths, Psychologist made a great impact too in psychological research (Griffiths Scale-Psychological Test). Numerous publications and discoveries were made at that time particularly when full-time Biochemist, Mr A H Tingey became a member of the research team.

In 1939, Mrs Burden gave further financial support to the Burden Neurological Institute which opened on 12 May 1939 when Professor F L Golla was appointed director of the Institute and Dr Grey Walter was in charge of the physiological research unit. These two people with their teams greatly contributed to the care and treatment of Mentally Handicapped people especially in connection with the treatment of epilepsy.

In 1948, Stoke Park Colony was absorbed in the National Health Service. Soon after Dr Norman became the director of Neuropathology at Frenchay Hospital and the rest of the research team was unfortunately disbanded and members moved to London and Oxford. However, in 1954, Dr Heaton-Ward, the new Medical Superintendent rekindled the spirits of his predecessors.

The links with the University and other local and national hospitals were gradually re-established and support was given by such eminent people as Professor Penrose, Professor Dent and locally Dr Eastham, who contributed greatly in the field of biochemistry and Dr F Lewis and recently Dr A McDermott in the field of chromosomal studies. Apart from research, the links with the community were first established in 1958 in the Central Health Clinic where in conjuction with Bristol Local Health Authority, the first assessment clinics were held by Dr A Heaton-Ward, Dr Lumsden-Walker and Dr J Jancar and others followed at the Gloucester Royal Hospital, Bush Training Centre, Yate and Kingswood Health Centres (Jancar, 1981).

In October 1961, at Hanham Hall Hospital, a new assessment unit was opened and 10 years later a multidisciplinary assessment unit was built at Stoke Park Hospital (Jancar, 1971).

In 1969, the Burden Trust instituted an annual award—'The Burden Research Medal and Prize' for outstanding research work in the United Kingdom and Ireland to commemorate Reverend Burden, the founder of Stoke Park Hospital and to encourage future research in the field of Mental Handicap. Nine people already received this coveted award.

In 1982 the post of Senior Lecturer in Mental Handicap at the Bristol University jointly with Stoke Park was established and the first holder of this post is Dr Yvonne V Wiley. At the same time, a Lecturer's post was also established and the first holder of the lectureship was the late Dr Graham Carter who tragically died. It is interesting to note that since research began at Stoke Park, 17 Books, 6 Chapters and over 350 Papers were published by the staff of the hospital group.

### HORTHAM COLONY

Hortham Colony was the first institution to be functionally designed and built as a complete colony and was opened on 29 April 1932. It was built by Bristol City Council and opened by the Minister of Health, Sir Edward Hilton-Young. The first patients were admitted from existing poor law establishments at Stapleton and Southmead. The first Medical Superintendent was Dr Walter Wyatt who came from Darenth Training Colony but who later left for Australia. His successor as Medical Superintendent was then Assistant Medical Officer, Dr J F Lyons. Dr Lyons showed by his outlook and leadership, an extraordinary ability to mould patients and staff into what he described as 'our family at Hortham'. I was privileged to witness this happy family when I joined Hortham Colony in 1948 as a Nursing Assistant.

In 1948 Hortham Colony joined with Brentry Hospital and became part of the National Health Service and the following units became part of the Hortham/Brentry Group: Rockhall School, Chasefield Laundry, Royal Fort Home and St Mary's Home in Painswick. Rockhall School and the House of Help were later replaced by Mortimer House in Clifton.

In 1955 Dr Lyons retired and Dr W Lumsden Walker succeeded him as Medical Superintendent. He continued with the philosophies of Dr Lyons and added new ideas. He continued to forge the links with the community and left in 1968 to take up a post at the Bristol Children's Hospital and child guidance. The gap was successfully filled by Dr Gordon-Russell who maintained and increased the momentum of progress until his recent retirement. The staff at Hortham and Brentry also produced very interesting publications (Upham and Roberts, 1982).

So far, we have looked at the institutions but we must note the very important role that the Social Services played in the provision of services for the Mentally Handicapped in Bristol, Bath and Somerset. I mentioned earlier, in the late part of the last century, various committees were looking at the variety of facilities and provisions to ameliorate the hardships of Mentally Handicapped people. Before and after the passing of the 1913 Mental Deficiency Act, a number of centres were opened and I will mention just a few of the most important ones.

In 1916 the Bristol Board of Guardians consented to the certification of the Stapleton Workhouse, for the reception of 50 Mentally Handicapped patients. Somerset County Council had taken over Yatton Hall in October 1919. In January 1920 temporary accommodation for the juvenile Mentally Handicapped was opened at Southmead Hospital. Marlborough House was opened by the Bristol Corporation in 1942. In 1963 the Bush Training Centre was opened and in 1965 Blackhorse Training Centre and Lanercost Road in November 1977. During the past two decades a number of residential hostels in Bristol and Bath were also provided.

Another very important contribution to the care of Mentally Handicapped people in Bristol was made by voluntary organisations. St Christopher's Private School opened in 1942 by Miss Grace who was trained at Stoke Park Hospital. Manor House, Frenchay, received the first Mentally Handicapped children in 1950. The Sheiling Curative School opened in Thornbury in 1952 and recently Priory Court at Hanham and Berwick Lodge, purchased from Southmead Health Authority.

I would also like to mention the Bristol Industrial Therapy Organisation opened in 1960, under the guidance of Dr Early, where a number of Mentally Handicapped, until quite recently, were helped and also employed.

A number of Bristol, Bath and Somerset Counsellors were active in the care and provision for Mentally Handicapped people and some outstanding Social Workers, eg, Morton, Penington and others who contributed greatly towards the welfare of the patients and their families. Mencap, Parents' Association, the Community Health Council and others played a very important part in providing a better future for the Mentally Handicapped.

### CONCLUSION

A recent wave of the 'magic' solutions for Mental Handicap, that everybody should be cared for in the community, thus resolving all the problems, is spreading through America, Scandinavia and Italy to this country—spearheaded by an Alliance with monetarists and so called 'normalisers'. Unfortunately the Alliance got the sums wrong. Community services, if run properly, are not cheap and 'normalisers' are not resolving the variety of mental and physical problems facing patients and families, but sometimes causing new ones. There is a gradual admission of failure by this Alliance and they are bringing a new idea of 'Advocacy' to cover the shortcomings of the philosophies they created.

In a recent report by the Social Services Committee—Community Care—to the House of Commons, the statement was made 'Any fool can close a hospital' and may I add to it that 'Not any fool can look after the Mentally Handicapped'.

I wish to conclude this address with the dictum from Cicero 'DUM TACENT CLAMANT'—'While silent they cry aloud'—while the Mentally Handicapped are suffering their disability in silence, they are crying aloud for your help and mine.

### REFERENCES

1. JANCAR, J. (1971) Assessment Unit for the Mentally Retarded. (A further observation). *Bristol med-chir. J.* **86**, 27.
2. JANCAR, J. (1972) Fifty Years of Brentry Hospital (1922–1972). *Bristol med-chir J.* **87**, 23.
3. JANCAR, J. (1974) The Gethin Shilling. *Bristol med-chir J.* **89**, 1.
4. JANCAR, J. (1981) Research at Stoke Park, Mental Handicap (1930–1980) The Supplement to Stoke Park studies. Henry Ling Limited, Dorset Press, Dorchester.
5. SHUTTLEWORTH, G. E. and POTTS, W. A. (1910) Mentally Deficient Children. Their Treatment and Training, third edition. H. K. Lewis, London.
6. UPHAM, B. C. AND ROBERTS, P. J. (1982) Hortham Hospital. Golden Jubilee—History of Change.

## Figures and Tables

**Figure 1 f1:**
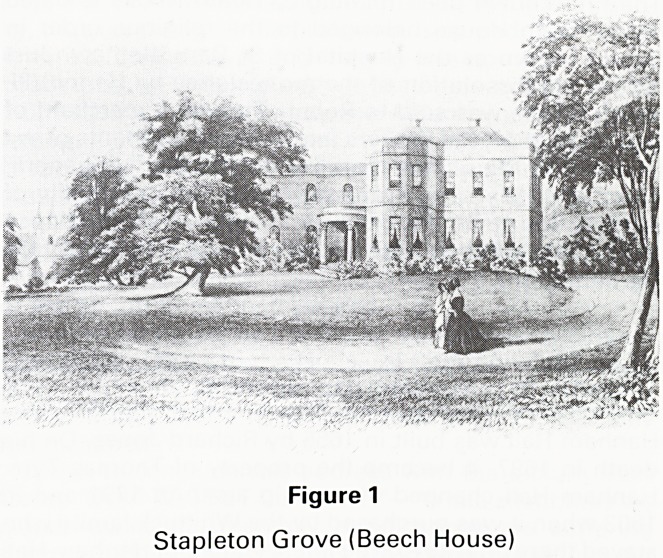


**Figure 2 f2:**